# Beyond TLR4 and Its Alternative Lipopolysaccharide (LPS) Sensing Pathways in Zebrafish

**DOI:** 10.3390/genes16091014

**Published:** 2025-08-27

**Authors:** Dara V. Grebennikova, Umesh K. Shandilya, Niel A. Karrow

**Affiliations:** Departmentof Animal Biosciences, University of Guelph, Guelph, ON N1G 2W1, Canada; dgrebenn@uoguelph.ca (D.V.G.); ushand@uoguelph.ca (U.K.S.)

**Keywords:** zebrafish (Zf), lipopolysaccharide (LPS) detection, TLR4-independent pathway, scavenger receptors (SRs), peptidoglycan recognition proteins (PGRPs)

## Abstract

Due to their evolutionary divergence from mammals, zebrafish (Zf, *Danio rerio*), which are frequently employed in biomedical research, provide a distinctive viewpoint on innate immune systems. The Toll-like receptor 4/myeloid differentiation factor 2/cluster of differentiation 14 (TLR4/MD-2/CD14) complex in mammals detects lipopolysaccharide (LPS), a crucial component of Gram-negative bacteria, and it causes potent inflammatory reactions through a Toll/interleukin-1 receptor domain-containing adapter-inducing interferon-β (TRIF)-dependent and myeloid differentiation primary response 88 (MyD88)-dependent pathways. However, key components of this system, such as a responsive TLR4 axis and a functional CD14 ortholog, are absent in Zf. The Zf species nevertheless reacts to LPS, which leads to research into other recognition systems. This review looks at a number of TLR4-independent processes in Zf, such as scavenger receptors (SRs) including scavenger receptor class B type 1 (SR-BI) and cluster of differentiation 36 (CD36), nucleotide-binding oligomerization domain-containing protein 1 (NOD1)-dependent cytosolic sensing, peptidoglycan recognition proteins (PGRPs), Complement Component 3 (C3), and caspase-1-like protein 2 (Caspy2)-mediated inflammasome activation. An alternative and flexible immune system that makes up for the lack of canonical TLR4 signaling is revealed by these mechanisms. Additionally, the discovery of *lymphocyte antigen 96* (*ly96*), an ortholog of MD-2 found in Zf, suggests evolutionary similarity; however, as it is only functional in artificial systems, it demonstrates minimal overlap with mammalian MD-2 activity. Knowing these pathways provides important information for studying inflammation, infection, and immunological modulation in vertebrates using Zf as a model. It also clarifies the evolutionary flexibility of innate immune recognition.

## 1. Introduction

The outer membrane of Gram-negative bacteria contains lipopolysaccharide (LPS) endotoxin, an essential and unique constituent that plays a key role in inducing inflammatory reactions across host species [1]. Once Gram-negative bacteria invade living organisms, specifically mammals, the Toll-like receptor 4/myeloid differentiation factor 2 (TLR4/MD-2) complex on surveillance cells can recognize bacterial components such as LPS with the help of cluster of differentiation 14 (CD14); these pattern recognition receptors (PRRs) set off a pro-inflammatory response via the activation of myeloid differentiation primary response 88 (MyD88)-dependent nuclear factor κ-light-chain-enhancer of activated B cells (NF-κB) that acts as a transcription factor regulating the production of cytokines through downstream signaling cascades [2].

Zebrafish (Zf, *D. rerio*) is a species of tropical freshwater fish that is popularly used as a model for research in areas such as genetics and development due to its high genetic similarity to humans (~70%) [3,4]. Additionally, thanks to its optical transparency, short generation time, and rapid development, the Zf model provides certain benefits that larger models such as mice cannot reproduce [5]. Current research highlights the Zf model’s substantial contributions to diverse areas of biomedical research, such as immunology, metabolism, cancer, toxicology, and neuroscience (Table 1).

Despite the genetic resemblance, the key pathway involved in recognizing LPS diverges from that of the mammalian pathway, with the Zf TLR4 paralogues (TLR4a and TLR4b) actually being found to negatively regulate the same MyD88-dependant NF-κB-dependent pathway, mainly due to the absence of CD14, which shuttles and presents the LPS to the TLR4/MD-2 complex [15]. Particularly when considering species-specific adaptations to microbial threats, this functional divergence highlights the Zf as a useful comparative model for examining various microbial detection techniques utilized by vertebrates and poses fascinating insight regarding the development of innate immunity across species. The Zf model also presents several limitations, including species-specific physiological differences, reduced organ complexity, and genetic redundancy due to whole-genome duplication [16]. Particularly in research involving intricate immune-organ interactions or diseases unique to humans, these features may hinder the direct translation of research findings to human biology.

The purpose of this review is to shed light on the functions of both conventional and alternative LPS recognition routes utilized by mammals, such as TLR4 signaling and the complement system, and evolutionary adaptations within the Zf species that enable it to recognize and mount an effector response against Gram-negative bacteria. This is of interest to deepen our understanding of species-specific innate immune systems, which may reveal hidden pathways and pharmacological targets outside of the normal TLR4 axis. Researchers can identify new immune pathways and regulators outside of the traditional TLR4 axis by contrasting traditional and alternative LPS recognition mechanisms across species, especially in Zf. These new immunomodulatory targets, like alternate receptors or signaling intermediates, present encouraging opportunities for creating more specialized and accurate methods to boost or inhibit immune responses during infections, inflammatory conditions, and immune-related illnesses, which could result in novel treatments with increased safety and effectiveness.

## 2. Established Pathways for LPS Recognition in Mammals

**Figure 1:**
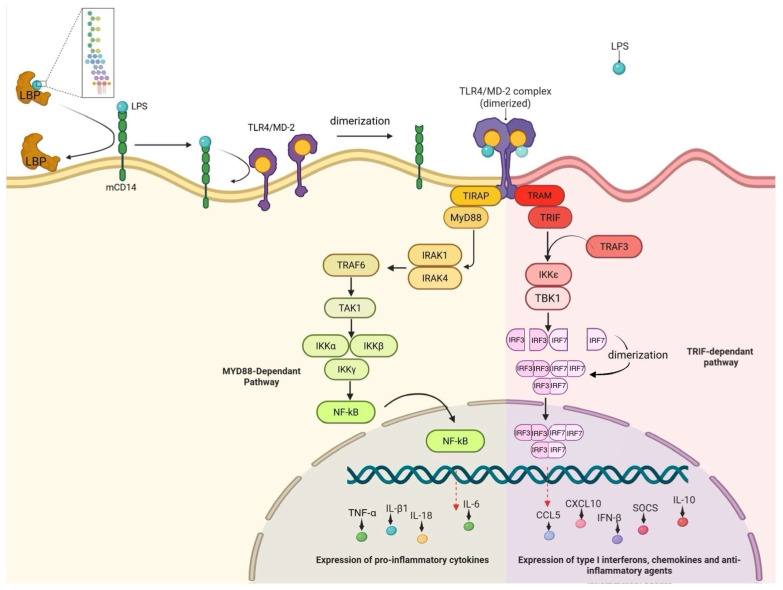
Mammalian canonical Toll-like receptor 4 (TLR4) signaling pathways: myeloid differentiation factor 2 (Myd88)-dependent (yellow) and Myd88-independent (pink) downstream signaling. Nuclear factor kappa-light-chain-enhancer of activated B cells (NF-κB) is activated, and pro-inflammatory cytokines are produced when the TLR4/myeloid differentiation factor 2/cluster of differentiation 14 (TLR4/MD-2/CD14) complex recognizes lipopolysaccharide (LPS), starting the MyD88-dependent pathway at the plasma membrane. However, when the MyD88-independent (Toll/interleukin-1 receptor domain-containing adapter-inducing interferon-β (TRIF)-dependent) route takes place following TLR4 incorporation into endosomes, it leads to interferon regulatory factor 3 (IRF3) and IRF7 activation and the generation of type I interferons. When exposed to Gram-negative bacterial LPS endotoxins, animals can generate both quick inflammatory/anti-inflammatory reactions and antiviral defenses because of this dual signaling pathway.

In mammals, the TLR4/MD-2 complex with the additional help of co-receptors constitutes the well-established and extensively studied LPS recognition mechanism. A soluble-LPS binding protein (LBP) is required to extract the LPS from the Gram-negative bacteria’s outer membrane and transport it to a CD14 molecule, expressed on the membrane of host surveillance cells, which include mucosal epithelial cells, tissue macrophages, dendritic cells, and mast cells [15]. Once LPS is bound to CD14, it is presented to MD-2 of the TLR4-MD-2 complex, also found on the cell membrane; this triggers the complex to dimerize, which facilitates surveillance cell activation [17]. The MyD88 pathway becomes activated through the recruitment of downstream adaptor proteins, including TIR-domain-containing adapter protein (TIRAP) and MyD88 itself, via interactions with its Toll/interleukin-1 receptor (TIR) domain [18]. Consequently, the transcription factor NF-κB becomes activated via several downstream activation steps (Figure 1), and this leads to the generation of pro-inflammatory cytokines, including interleukin (IL)-1β, IL-6, IL-18, and tumor necrosis factor alpha (TNF-α), that promote a defensive innate immune response to the infection [18]. A recent study used a single-step fluorescent assay that mimics the sequential in vivo route to successfully illustrate the fundamental elements of the TLR4-LPS complexation process [19]. By using immobilized recombinant human TLR4 and magnetic separation, these authors demonstrated that LPS binding resulted in the quick and precise development of the TLR4-MD-2 complex in the presence of CD14 and MD-2 [19].

In addition to the MyD88-dependent signaling, TLR4 also initiates a unique Toll/interleukin-1 receptor domain-containing adapter-inducing interferon-β (TRIF)-dependent pathway after being internalized into endosomes [20,21]. Endocytosis is facilitated via the co-receptor CD14 following the detection of LPS [22]. Independent of MyD88, TLR4 initiates a signaling cascade within the endosomal compartment by enlisting the adaptor proteins TRIF-related adapter molecule (TRAM) and TRIF [21]. TRIF interacts with the ubiquitin ligase tumor necrosis factor receptor-associated factor 3 (TRAF3) to activate the inhibitor of kappa-B (IκB) and kinase TANK-binding kinase 1 (TBK1), as well as the inhibitor of nuclear factor kappa-B kinase subunit epsilon (IKKε) [21]. TBK1 phosphorylates interferon regulatory factor 3 (IRF3) and interferon regulatory factor 7 (IRF7), promoting their activation, homodimerization, or heterodimerization, allowing them to undergo nuclear translocation where they act as transcription factors [21,23,24]. This leads to the production of chemokines like C-C motif chemokine ligand 5 (CCL5) and C-X-C motif chemokine ligand 10 (CXCL10) and type I interferons like IFN-β (Figure 1),which are essential for the development of adaptive immunity and antiviral responses [21]. Furthermore, delayed NF-κB activation, the regulation of cell death pathways, and the synthesis of anti-inflammatory cytokines like IL-10 and suppressors of cytokine signaling (SOCS) are all facilitated by TRIF signaling [21].

## 3. Early Studies and the Mystery of Certain Teleost Fish

Unlike most fish genomes, which have lost the *TLR4* gene, Zf, as well as a handful of other species such as the common carp (*Cyprinus carpi*) and Atlantic cod (*Gadus morhua*), retained a copy [25,26]. When comparing the *TLR4* genes between the Zf and mammals, Sullivan et al. stated that Zf *TLR4* paralogues, of which there are two, share roughly 67% identity with each other, but only around 35–38% identity with human and mouse *TLR4*, especially in their extracellular domains, in the region where MD-2 protein binds in humans [27]. However, despite lacking a functional mammalian-like TLR4, Zf still show evidence of responding to LPS [28,29]. Common Gram-negative bacteria that are reported to be infecting the Zf include *Aeromonas veronii*, a bacterium found in both the gut and freshwater, members of the *Vibrio* family, also commonly found in aquatic environments, and *γ-Proteobacteria* [30,31,32]. By exposing Zf larvae to varying concentrations of bacterial LPS (*Escherichia coli* 0111:B4, *E. coli* 055:B5) and tracking survival rates, Novoa et al. showed that concentrations of *E. coli* 0111:B4 LPS ranging between 0 and 50 µg/mL resulted in low or no significant mortality, while at concentrations between 150 and 200 µg/mL, death rates skyrocketed, demonstrating that LPS induces a physiological response [28]. Both *E. coli* strains are commonly used in immunological studies and are often associated with contaminated freshwater environments. The LPS of *E. coli* O111:B4 and O55:B5, two powerful endotoxin-producing serotypes, aggressively stimulate immunological responses. *E. coli* O111:B4 LPS often causes faster and more severe inflammatory consequences, but *E. coli* O55:B5 LPS also produces strong, albeit slightly different, immune activation profiles. Both are employed in studies to investigate inflammatory processes and bacterial pathogenesis. Additionally, if larvae were pretreated with a sublethal concentration of LPS, they exhibited tolerance when treated once more, demonstrating that Zf may establish an immunological response to LPS, albeit through methods distinct from the mammalian TLR4 pathway [28]. Additionally, research by Wang et al. further demonstrated that Zf exposed to a lethal dose of *Pseudomonas aeruginosa* LPS endotoxin, a natural inhabitant of freshwater environments, experienced severe physiological stress, reinforcing the conclusion that Zf are able to respond to LPS without recognition via TLR4 [29]. Additionally, Wang et al. pretreated larval Zf with 6000 μM sodium butyrate (NaB) at 72 h post-fertilization (hpf) by replacing the egg water in each well of a 96-well plate with NaB solution [29]. Afterwards, at 96 hpf, the NaB solution was replaced by the LPS, allowing for environmental exposure [29]. The larvae pretreated with NaB had a higher survival rate than those untreated (*p* = 0.012), suggesting that NaB provided a protective effect against LPS-induced mortality [29].

In an earlier study, Sepulcre et al. proposed that Zf might use a TLR4-independent pathway to recognize LPS, as their TLR4 paralogues (TLR4a and TLR4b) were found to negatively regulate the MyD88-dependent NF-κB pathway, the same pathway that drives the immune response in mammals [15]. By employing functional assays to transfect human cells with constructs that expressed Zf TLR4 paralogues and NF-κB-luciferase reporter that fluoresces upon NF-κB activation, they discovered that TLR4a suppressed NF-κB activation, even in the absence of LPS, but not TLR4b [15]. Furthermore, by engineering variants of TLR4b, TLR3 and TLR4 that lack their extracellular domains, dubbed ZfTIR4b, ZfTIR3, and ZfTIR9, these authors showed that ZfTIR3 and ZfTIR9 activated NF-κB when overexpressed; however, ZfTIR4b slightly reduced NF-κB activity, suggesting that it might act as a negative regulator [15].

Notably, the TIR domain of both Zf TLR4 paralogues has a non-conservative sequence variant in which a proline that is typically conserved in other species is coded by an alanine residue. It is well recognized that this proline residue is necessary for TLR4 activation, as when the proline found in mouse TLR4 was replaced with histidine, the new mutant was rendered non-functional [15]. This particular histidine mutation in mouse TLR4 impairs the receptor’s functionality and its capacity to communicate subsequent inflammatory reactions. By creating a similar mutation in the Zf *TLR4b*, replacing the proline with histidine, the mutation outcome was comparable to the loss-of-function mutation in mouse TLR4 [15].

An additional study tested for co-expression of Zf TLR4a and TLR4b in human and Zf cells; however, no antagonistic or synergistic effects on NF-κB activation were observed [27]. These authors demonstrated using chimeric proteins that Zf TLR4 extracellular domains do not react to LPS, but that when combined with mouse extracellular domains, their transmembrane and intracellular domains can activate NF-κB [27]. These findings imply that Zf TLR4s may identify other ligands or function as co-receptors instead of reacting to LPS, in contrast to previous research that suggested their TIR domains block NF-κB [27].

Collectively, these results point to the evolutionary flexibility of TLR-mediated signaling pathways across species, indicating that, whereas Zf TLR4 paralogues do not participate in classical LPS recognition, they do have alternate, potentially regulatory roles in innate immunity.

## 4. Alternative LPS Detection Mechanisms in Zebrafish

**Figure 2:**
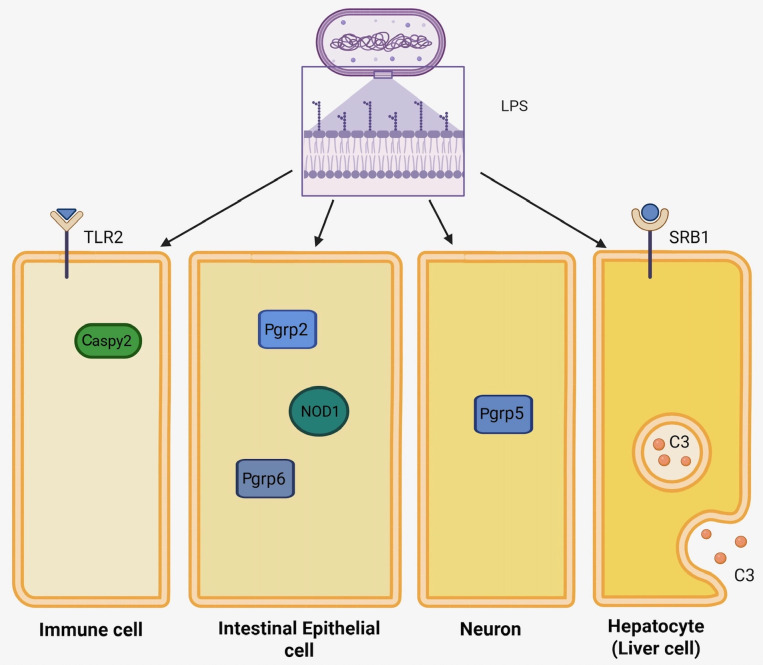
Diagram of the potential alternative LPS detection mechanisms in zebrafish. Four simplified cells with their respective elements. Toll-like receptor 2 (TLR2) and caspase-1-like protein 2 (Caspy2) are found in the membrane and cytosol of the macrophage/neutrophil compartment, respectively. Peptidoglycan receptor protein 2 (PGRP2), peptidoglycan receptor protein 6 (PGRP6), and nucleotide-binding oligomerization domain-containing protein 1 (NOD1) are intracellular components of intestinal epithelial cells. Peptidoglycan receptor protein 5 (PGRP5) is found intracellularly in neuronal cells. The hepatocyte compartment contains membrane-bound scavenger receptor class B type 1 (SR-B1) and intracellular complement component 3 (C3) that is being exocytosed.

With TLR4s of unknown function, researchers hypothesize that Zf utilize a TLR4-independent pathway to recognize organisms containing LPS endotoxins. Other innate immune receptors and downstream effectors that can trigger an inflammatory response may play an alternative role in LPS recognition and will be discussed below (Figure 2).

### 4.1. Caspases

Caspases, a family of endoproteases, play a critical role in cell regulation and are classified into two main groups, focusing either on apoptosis or inflammation [33]. They are activated upon cellular stress, such as infection, with some responding to LPS. For example, mouse caspase-11 is found to be activated upon detecting intracellular LPS and induces IL-1ß production; this has been referred to as the noncanonical LPS recognition pathway [34,35]. Caspases differ from each other based on their various N-terminal prodomains, with caspase-11 (mouse) having a Caspase Recruitment Domain (CARD) at its N-terminus that allows it to mediate interactions with adaptor proteins and is essential for recognizing intracellular LPS [36,37]. Recognition of intracellular LPS triggers caspase-11 oligomerization and activation without the need for additional adaptor proteins. This direct interaction with LPS is essential for sensing cytosolic LPS and initiating the alternative inflammasome pathway [37]. In humans, caspase-4 and caspase-5 serve as functional orthologs of mouse caspase-11, sharing significant sequence similarity and a conserved CARD that enables them to similarly detect and respond to intracellular LPS [38].

With mammalian caspases showing TLR4-independent ways of recognizing LPS endotoxins, similar hypotheses were also applied to Zf, specifically caspase-1-like protein 2 (Caspy2), a Zf-specific inflammatory caspase. Caspy2 contains a pyrin domain (PYD) at its N-terminus, which also functions as a protein–protein interaction module, and this domain was found to be homologous to that of human Cryopyrin/PYPAF1 (46% similarity), a pyrin domain-containing NOD-family protein 10 [39]. Murine CARD and Zf PYD both belong to the death domain superfamily of signaling domains [40]. In mice, CARD activates inflammasome assembly by binding to LPS [41]. In Zf, research supports that the PYD found in Zf Caspy2 plays a critical role in mediating intracellular LPS binding, resulting in caspy2 oligomerization and noncanonical activation of the inflammasome [41]. Additional studies have shown that Caspy2 oligomerizes and is activated when it directly attaches to LPS without the aid of adaptor proteins or surface receptors, unlike TLR4 receptors, which require the aid of MD-2 and CD14 [41]. An inflammatory form of programmed cell death called pyroptosis is then initiated, promoting the maturation and release of pro-inflammatory cytokines like IL-1β [41]. This mechanism reflects an evolutionarily conserved alternative strategy by which Zf and mammals detect and respond to Gram-negative bacterial infections. A later study by Zhuang Wang et al. used a rapid clustered regularly interspaced short palindromic repeats/CRISPR-associated protein 9 (CRISPR/Cas9)-based genetic screening method to directly knock out (KO) genes linked to pyroptosis, and these authors were able to demonstrate that targeting Zf Caspy2 and Gasdermin Eb (GSDMEb) protected larvae from lethal LPS-induced septic shock [42].

### 4.2. Peptidoglycan Recognition Proteins (PRGPs)

As their name suggests, peptidoglycan recognition proteins (PGRPs) can recognize peptidoglycan structures of both Gram-positive and Gram-negative bacteria. These PRRs are highly conserved across species from insects to mammals, where they trigger an immune response to resist infection [43]. Peptidoglycan structures vary among bacteria, with different amino acid cross-linkages, allowing them to be distinguished by different PGRPs to tailor the immune response based on the specific bacterial threat [43].

According to current research, Zf possess four *PGRP* genes, with only three having been cloned and characterized [43]. The fourth gene has been identified from an expressed sequence tag (EST), but its full-length cDNA remains uncharacterized [44]. PGRP2, PGRP5, and PGRP6 are the three PRRs that have been established to support the immune response [34]. They all share a conserved N-acetylmuramyl-L-alanine amidase domain, which is essential for their bactericidal activity and ability to hydrolyze peptidoglycan, because of their enzymatic activity [44]. PGRP2, being mostly highly expressed in the intestine, liver, and eggs, and identical to mammalian peptidoglycan recognition protein 2 (PGLYRP2), plays a crucial part in gut immunity [45]. PGRP5, on the other hand, is extensively expressed in the brain, eyes, and muscles, indicating a possible neuroimmune role [45]. These discrepancies demonstrate that their shared functionality, evolutionary lineage, tissue distribution, and putative physiological roles vary [46]. Correspondingly, Li et al. found that PGRP-5 was significantly upregulated following *E. coli* infection and is strongly expressed in the brain and muscle tissue of Zf embryos. Little research has been conducted on the specific function of PGRP6; however, it has been established that it is mainly expressed in the intestines and eyes [45]. From current research, PGRP5 holds the most promise in LPS recognition. Li et al. showed altered neurobehavior, reduced antioxidant enzyme activity, and changes in the expression of pro- and anti-inflammatory cytokines when *PGRP5* was knocked down [45]. Specifically, a knockdown via vivo-morpholinos reduced *PGRP5* transcriptomic expression by roughly 80% as indicated by qPCR, resulting in a decrease in the pro-inflammatory cytokines IL-1β, IL-6, and TNF-α, as well as an increase in anti-inflammatory cytokine transforming growth factor β (TGF-β) [45].

Although Zf PGRPs are primarily recognized for their specificity to peptidoglycan, emerging evidence suggests a potential indirect involvement in modulating immune responses to LPS. A 2016 study by Iatsenko et al. concluded that in insects such as fruit flies (*Drosophila melanogaster*), PGRPs can detect and differentiate Gram-negative and Gram-positive bacteria due to the diaminopimelic acid (DAP)-type or lysine (Lys)-type peptidoglycan, found commonly, but not exclusively, in the respective bacteria [47]. Wang et al., found that exposure to LPS increased *PGRP* gene expression in Zf larvae, despite most of the literature suggesting that PGRPs are specific for the detection of peptidoglycan and do not directly bind to or neutralize LPS [29]. This data does not directly demonstrate that PGRPs recognize LPS as a ligand, but it does raise the possibility of a connection between PGRP-mediated immune responses and LPS-induced inflammation.

Overall, even though Zf PGRPs are mainly recognized for their ability to detect peptidoglycan, their elevated expression following exposure to LPS points to a potentially more diverse function in immune regulation. PGRPs, particularly PGRP5, seem to trigger inflammation and may be a component of a larger LPS-responsive network, even though they might not directly bind LPS.

### 4.3. Scavenger Receptors (SRs)

Mostly present on endothelium and certain epithelial cells, as well as phagocytic cells like macrophages, scavenger receptors (SRs) are a diverse class of PRRs with the ability to identify and bind to a wide range of negatively charged polyanionic ligands [48]. With LPS being a polyanionic ligand, current research supports that specific classes of SRs, class A (SR-A) and class B (SR-BI and SR-BII), for example, both play a protective and regulatory role in response to LPS-associated infections in mammals [49,50]. Through both MyD88-dependent and MyD88-independent mechanisms, TLR4 activation in response to LPS upregulates the SR-A, Macrophage Receptor with Collagenous Structure (MARCO), and SR-A [49]. Notably, LPS has been shown to directly bind to SR-A and MARCO via their collagenous domains and aid in eliminating LPS from the bloodstream [51]. One study demonstrated reduced LPS absorption and heightened inflammatory responses after blocking or genetically deleting these SRs using a mouse model [49]. Using two groups, Wild-Type (WT) and KO, both groups were subjected to LPS at a low dose (25 µg per mouse) and a high dose (6 mg per kg of body weight) [49]. At the low dose, the WT group upregulated MARCO and SR-A, while the KO group experienced no regulation [49]. After the high dose, the WT group displayed heightened inflammatory responses and reduced survival, while the KO group had improved survival, higher B cell activation, increased IL-10 production, and more anti-LPS antibodies following a two-step LPS challenge [49]. These results imply that the SRs MARCO and SR-A are able to assist in recognizing and eliminating LPS, although when challenged by high LPS concentrations, their overexpression may aggravate inflammatory damage [49]. By strengthening anti-inflammatory and adaptive immune responses, deleting these receptors lowers dangerous inflammation and increases survival [49].

Supplementary research by Baranova et al. demonstrated that by controlling cytokine synthesis and immune cell activation, the Class B SRs aided in limiting excessive inflammatory damage [50]. It has also been demonstrated that SR-BI and SR-BII affect macrophage responses by increasing anti-inflammatory signals and decreasing pro-inflammatory cytokines such as TNF-α and IL-6 [50]. Following LPS exposure, mice lacking these receptors showed greater tissue damage and inflammation, suggesting that SR-BI and SR-BII have a protective role by regulating immune activation and averting excessive inflammation during bacterial endotoxin challenges [50].

Research on teleost fish supports the involvement of SRs in the response to LPS. Meng et al. reported the first gene cloning and functional characterization of the class A SR from *Dichotomyctere (D.) nigroviridis* (referred to as *Tetraodon nigroviridis* in the original study), subsequently called TnSR, which is homologous to SR class A member 5 (SCARA5) in humans and mice, a type II transmembrane protein [52]. Although *TnSR* was originally cloned from *D. nigroviridis*, its Zf ortholog shares a high degree of structural and sequence homology [53]. Unlike most other teleost fish, including *D. nigroviridis*, Zf possess TLR4 paralogs, making them a practical system for investigating alternative LPS recognition pathways [54,55]. By injecting Zf embryos with 2 µg of LPS (*E. coli* O55:B5) and assessing the expression of *TnSR* using RT-PCR, the authors were able to demonstrate that *TnSR* expression increased in most tissues compared to untreated fish, with significant increases in the spleen (82.3-fold increase), muscle (37.8-fold increase), brain (12.4-fold increase), and kidney (3.8-fold increase) [52]. The *E. coli* strain used is a frequently used laboratory strain, with its LPS (obtained from Sigma-Aldrich) serving as a standard endotoxin for experimental immune stimulation. These authors also found that LPS caused dose-dependent activation of NF-κB using a Zf embryo model and NF-κB dual luciferase reporter system [52]. Even below baseline levels, co-injection with LPS and TnSR (pcDNA60-TnSR) dramatically attenuated NF-κB activation, indicating that TnSR has a negative regulatory function during inflammation [52]. Their subsequent mechanistic research also showed that TnSR competitively bound to TNF receptor-associated factor 2 (TRAF2), a crucial adaptor protein in the TNF-α signaling pathway, to prevent TRAF2 from increasing inflammation and consequently suppress NF-κB activation [52]. Competitive interaction between TnSR and TRAF2 was confirmed by this group when TRAF2 overexpression reversed the inhibitory impact of TnSR on NF-κB activation [52]. Furthermore, TnSR inhibited TNF-α-induced NF-κB activation, confirming its role as a negative regulator in teleost fish inflammatory signaling pathways [52].

A second study by Liu et al. determined that a Zf homolog of mammalian cluster of differentiation 36 (CD36), a SR of the class B SR family (51.6% amino acid identity with human CD36), is able to directly bind to both Gram-positive and Gram-negative bacteria as indicated via Western blotting and fluorescent labeling [56]. This implies that CD36 contributes to bacterial identification in the Zf even though the precise binding mechanism is unknown [56]. Since Zf do not have certain common PRRs, like mammalian CD14, the SRs like CD36 may play a special immune defensive role in this fish species [56].

By mediating pathogen recognition, modifying inflammatory signaling, and impacting host survival outcomes, SRs play important roles in controlling immune responses to LPS in mammals and lower vertebrates. Additional research on crustaceans has discovered that SR-BII, a type III membrane PRR, recognizes LPS and activates the immune deficiency (IMD) pathway [57]. In insects and some arthropods, the IMD pathway, which is a widely conserved NF-kB immunological signaling pathway, controls the organisms’ antibacterial defense reaction [58]. This study found that in response to bacterial infection, SRBII expression increased, and knockdown of *SRBII* led to heightened bacterial proliferation and reduced survival in Japanese tiger prawn (*Marsupenaeus japonicus*) [57]. Functionally, the intracellular C-terminal domain of SRBII, which contains a cryptic Receptor-Interacting Protein Homotypic interaction motif (RHIM)-like motif, interacts with IMD to facilitate RELISH nuclear translocation and pathway activation, whereas its extracellular domain binds to LPS [57]. Additionally, LPS-induced IMD signaling and diptericin production are enhanced when shrimp *SR-BII* is expressed in Drosophila Schneider 2 (S2) cells [57].

In summary, several SRs are key innate immune receptors that bind LPS and affect inflammatory outcomes, especially the class A (SR-A, MARCO) and class B (SR-BI, SR-BII, CD36) SRs. These SRs aid in the removal of LPS and regulate the generation of cytokines in mammals, but they can also worsen inflammation when LPS levels are elevated. Homologues like TnSR and CD36-like SRs control inflammation in Zf and other teleost by binding bacteria and suppressing NF-κB, respectively. Similarly, by binding LPS and initiating subsequent immunological signaling, SR-BII in crustaceans triggers the IMD pathway. These cross-species results demonstrate the conserved yet context-dependent functions of SRs in regulating inflammation and boosting immunity when exposed to LPS.

### 4.4. Nucleotide-Binding Oligomerization Domain (NOD) Genes

The nucleotide-binding oligomerization domain (NOD) genes code for a family of intracellular PRRs called NOD-like receptors (NLRs) [59,60]. Through their ability to identify microbial compounds, such as muramyl dipeptide (MDP) from peptidoglycan, within cells, and initiate immunological responses, these PRRs are essential components of the innate immune system [61]. According to computational modeling, there are approximately 2000 Zf NLR genes; however, laboratory strains reveal that there are, impressively, more likely about 400 known genes [62]. Out of those hundreds of genes, five belonging to the NLR-A subfamily have been found to be the most closely related to mammalian *NOD* genes [63]. These five Zf genes include *NOD1*, *NOD2*, *NOD3*, *NOD4*, and *NOD5*, suggesting that they may also play a conserved role in bacterial peptidoglycan recognition [61,63,64,65]. The roles of the remaining NLRs seem to have evolved and are now more distantly connected; however, many appear to continue to be linked to important immune-related functions, such as regulating inflammation, antiviral defense, and wider cellular reactions [63].

Recent research has implicated NLRs in detecting LPS endotoxins. In mammals, NOD1 detects iE-DAP, frequently found in Gram-negative bacteria, while NOD2 detects MDP from peptidoglycans frequently associated with Gram-positive bacteria, although both can be interchangeable between the two groups of bacteria [66,67]. Recent research has demonstrated that NOD1 may directly detect LPS in Zf and other fish species, activating the NF-κB signaling pathway and causing the release of pro-inflammatory cytokines. According to Bi et al., NOD1 functions as a cytosolic PRR sensor in teleost fish that directly detects LPS and causes NF-κB activation and the production of inflammatory genes [60,67]. These authors demonstrated through overexpression and knockdown studies that NOD1 regulates the immune response to LPS; NOD1 knockdown decreased the response, whereas overexpression increased it [60,67]. The consequences of a NOD1 knockdown lead to a reduction in an inflammatory response, with lower NF-κB activity [60,67]. These results support the hypothesis that fish use an alternate TLR4-independent route involving NOD1 to detect LPS and respond to Gram-negative pathogens.

Additional research by Bi et al. found that NOD1 directly binds to LPS via pulldown and ELISA assays, even stronger than iE-DAP, suggesting that NOD1 is a receptor for LPS in Zf [60]. Furthermore, by regulating Cluster of Differentiation 44 (CD44) expression and triggering the phosphoinositide 3-kinase (PI3K)–Akt (protein kinase B) signaling pathway, a crucial mediator for cell survival, it is has been demonstrated that NOD1 plays a crucial role in Zf larval survival [68]. CD44, a hyaluronan receptor and transmembrane adhesion molecule, is known to trigger the PI3K-Akt pathway, which is linked to cell survival in a variety of cell types [68]. While overexpression of CD44 restored the phenotype, NOD1 deletion disrupted this pathway and decreased viability [68]. These results imply that NOD1 participates in broader cellular functions like survival signaling in addition to its traditional function in bacterial detection. Although Y. W. Hu et al. did not specifically investigate the capacity of NOD1 to detect LPS, their findings lend credence to the idea that NOD1 may function as a multipurpose receptor in Zf, aiding in both immunological defense and the control of intracellular survival pathways [68]. However, more research on Zf NLRs is needed to expand our understanding of alternative immune recognition techniques.

### 4.5. Toll-like Receptor 2 (TLR2)

TLR2 is another essential PRR that can identify a wide variety of microbial products. To broaden its ligand repertoire, TLR2 frequently forms heterodimers with TLR1 or TLR6 [69]. In mammals, TLR2 is most well-known for its capacity to identify elements from Gram-positive bacteria, including bacterial lipopeptides, peptidoglycan, and lipoteichoic acid [69]. In addition to having different ligands, TLR2 and TLR4 also differ in their elicited cytokine responses, with TLR4 activation usually resulting in a stronger T helper 1 (Th1)-type inflammatory response, whereas TLR2 activation stimulates both Th1 (IFN-γ, IL-2, TNF-α) and Th2 cytokines (IL-4, IL-5, IL-10, IL-13) [70].

In Zf, TLR2 is a highly conserved protein that functions similarly to its mammalian equivalent [71]. It responds strongly to bacterial lipopeptides and plays an essential role in host defense [71]. For example, one study specifically tested for TLR2’s responsiveness to *Mycobacterium marinum*, a bacterium commonly found in freshwater [71]. By using a Zf TLR2 mutant (*TLR2^sa19423^* mutant, homozygous (^−/−^), heterozygous (^+/−^), and WT (^+/+^) as control), these authors were able to demonstrate that the mutants with non-functional TLR2 exhibited increased susceptibility to infection, decreased expression of important inflammatory genes such as those involved in chemokine signaling, and decreased granuloma formation [71].

Despite the lack of direct evidence for TLR2-mediated LPS recognition in Zf, evidence from other species, such as mice, suggests the existence of potential compensatory pathways or receptor crosstalk mechanisms within the innate immune system [72]. For example, one study examined the response to atypical LPS from *Ochrobactrum* (*O*.) *intermedium* human HEK293T cells expressing TLR2 and TLR4/MD-2/CD14, as well as murine macrophage cell lines and primary peritoneal macrophages [73]. The study found that both TLR2 and TLR4 were capable of recognizing these atypical LPS structures—variations of LPS that typically induce weaker inflammatory responses compared to *E. coli* LPS—demonstrating that multiple TLRs can detect noncanonical LPS molecules from bacteria such as *O. intermedium* and *Brucella abortus*, which normally elicit reduced immune activation [73]. Neither bacteria are typical freshwater species, although they can occasionally be detected in aquatic environments, and were chosen in the study primarily for their structural properties. *O. intermedium* LPS elicited much lower levels of macrophage pro-inflammatory cytokines (IL-6, IL-12, and TNF-α) using *TLR2* and *TLR4* mutant mice, and a 100-fold higher dose was required to elicit a comparable immunological response to *E. coli* LPS [73]. Furthermore, both TLR2 and TLR4 mutant macrophages produced fewer cytokines, suggesting that *O. intermedium* LPS interacts with both receptors [73]. Human Embryonic Kidney (HEK) cells expressing either TLR2/TLR6 or TLR4/CD14/MD-2 were also used to further validate this dual reliance [73]. *O. intermedium* LPS activated NF-κB in both cell lines, although much less strongly than *E. coli* LPS, and in some cases, these structures even encouraged the production of TLR2/TLR4 heterodimers [73]. Given that TLR2 can aid in the recognition of LPS-like molecules under specific structural conditions, it is speculated that these findings highlight the surprising versatility of innate immune recognition and support the theory that atypical LPS may activate additional or different TLRs in a species-specific or context-dependent manner [73]. However, functional studies consistently demonstrate that TLR2 is not necessary for the canonical immunological response to LPS, and TLR2-deficient Zf do not display decreased LPS-induced signaling or inflammation [74]. This implies that, unlike mammals, which may use TLR2 as a co-receptor or fallback mechanism for identifying unusual LPS, Zf rely on alternate, TLR2-independent mechanisms for LPS detection and immunological activation.

Together, these results imply that zebrafish TLR2 does not seem to be directly engaged in LPS sensing, although it maintains conserved roles in host defense and bacterial detection. Zf may also use unique, yet undiscovered, receptors or pathways to detect LPS, which reflects a gradual evolutionary difference in innate immune responses.

### 4.6. Complement Protein C3

The complement system is a network of plasma and tissue-associated proteins that make up a key effector component of the innate immune system [75]. Many of these proteins propagate throughout the body in an inactive (zymogen) state, yet when triggered via an infection or injury, they become activated in a sequential enzymatic cascade [75]. Among them, Component 3 (C3), a large glycoprotein primarily produced by the liver, functions as the complement cascade’s central initiation point [76]. Upon activation, C3 is enzymatically cleaved into two fragments: anaphylatoxin C3a, which acts as a chemoattractant that draws leukocytes to the infection site and also triggers inflammation, and C3b, which covalently binds to the surface of pathogens, acting as an opsonin tagging them for destruction [75]. These events increase the effectiveness of pathogen removal while also boosting the complement response, which ultimately results in the creation of the membrane attack complex (MAC) that can directly lyse invasive microorganisms [75].

Zf C3 is a functional homolog of mammalian C3, similarly playing a role in opsonization and defense against pathogens [77]. Currently, limited research supports the hypothesis that C3 has a direct role in LPS recognition by Zf. Research suggests that instead of directly binding LPS, C3 is thought to be secondarily activated following initial pathogen detection [78]. It then opsonizes the pathogen, recruits effector cells to participate in the immune response, and initiates formation of the MAC [78]. For example, Forn-Cuní et al., concluded that after Zf were exposed to LPS, certain *C3* genes demonstrate increased expression, indicating a role in inflammatory response [79]. Another study concluded that Zf C3 can bind to both Gram-negative and Gram-positive bacteria after generating and purifying recombinant Zf C3 protein and incubating it with various bacterial strains [80]. Using immunodetection tests, these authors evaluated C3 binding and demonstrated that it could adhere to the surfaces of both kinds of bacteria [80].

An additional study by Wang et al. demonstrated evidence of the functional significance of C3 in Zf innate immunity by looking into the complement system maturation and response to LPS in the early stages of life [81]. Their results demonstrated that *C3* and other complement genes are expressed as early as two hours after conception, and their expression increased continuously after hatching [81]. Notably, *C1r/s* from the classical complement pathway, as well as Complement 4 (C4) and Mannan-binding lectin-associated Serine Protease (MASP) from the lectin-binding complement pathway, did not exhibit comparable responsiveness to LPS stimulation, but *C3* and *factor B* (*Bf*), both essential to the alternative pathway, showed notable upregulation in response to LPS challenge soon after hatching [81]. This implies that the alternative pathway plays a major role in reacting to LPS at this early point in development and becomes functionally active before the other complement arms [81]. Despite its potential inability to directly identify LPS, C3’s early activation after exposure suggests that, if pathogen-associated molecular patterns (PAMPs) are identified, it plays a crucial part in enhancing the immune response [81]. These findings highlight the crucial effector role of Zf C3 in innate immunity, but its potential to bind to LPS remains to be determined.

### 4.7. Updated Perspectives on Zf LPS Detection Mechanisms

As previously mentioned, due to several underlying factors such as the absence of key co-receptors MD-2 and CD14, the Zf inflammatory response to LPS endotoxins differs from that of mammals, and it is attenuated in comparison. However, a recent study challenges this established understanding by introducing a novel perspective [82]. By performing BLAST v2 analyses of the Zf genome and transcriptome, beginning with the human MD-2 protein sequence and progressively comparing across species from mammals to amphibians and fish, the authors were able to identify a Zf MD-2 ortholog coded by the *lymphocyte antigen 96* (*ly96)* gene, the same gene that codes for MD-2 in mammals, suggesting that a functional TLR4/MD-2 complex may exist in Zf [82]. Accordingly, the authors designate the Zf MD-2 protein “ly96” in reference to its encoding gene, distinguishing it from its mammalian counterpart [82]. According to the same study, Zf ly96 has the ability to mediate the TLR4 response; more precisely, TLR4ba rather than TLR4bb, but only when co-transfected with mammalian (human, mouse, opossum) *CD14* [82]. These authors investigated the activation of TLR4ba and CD14 without transfecting *ly96*, which showed no response to LPS, confirming that ly96 was necessary for TLR4ba activation rather than CD14, which remains unidentified in Zf [82]. This work further emphasizes the functional divergence and evolutionary conservation of LPS-sensing pathways between mammals and Zf [82]. These findings additionally challenge the earlier theories which emphasize the lack of important co-receptors in Zf and imply that, in spite of evolutionary divergence, Zf still have elements of a functional TLR4/MD-2 signaling complex [82]. However, the fact that these experiments required human CD14 suggests that the Zf counterpart of this co-receptor, if any, has an unknown function, and that Zf relies on another mechanism for LPS detection [82]. The evolution of innate immunity and the molecular adaptations that allow Zf to recognize and react to bacterial endotoxins through both conserved and unique pathways can now be further investigated thanks to this discovery [82]. This additionally rebuts the idea that there is a presence of inhibitors blocking the functionality of a Zf TLR4 pathway, as the study unequivocally shows that the absence of signaling is caused by the absence of crucial elements, namely MD-2 (ly96) and CD14. Since TLR4ba signaling is fully restored when these components are present, it is doubtful that any conceivable inhibitory molecule could explain the inactivity because it would likely still have an effect even when the restored components are present.

## 5. Implications and Evolutionary Perspective

Even with the recent discovery of a Zf *ly96* that codes for a low-functional MD-2, it remains widely accepted that the Zf uses TLR4-independent pathways for the promotion and mediation of an inflammatory response to LPS endotoxins. Sepulcre et al. theorize that due to continuous exposure to bacteria in aquatic habitats, evolutionary pressures may have favored mechanisms that suppress excessive LPS-induced inflammation in teleost species, as shown in their Zf study that TLR4 negatively impacts the MYD88 pathway. Persistent immunological activation at high microbial concentration could be detrimental, possibly resulting in chronic inflammation and tissue damage [15]. Consequently, Zf seem to have evolved defenses against excessively violent inflammatory responses, which aid in immunological homeostasis [15]. This adaptation shows how to balance identifying microbial threats while avoiding harmful immune responses. To draw a parallel with mammals, it has been speculated that bats (order *Chiroptera*) have evolved alongside viral pathogens and are able to harbor and tolerate them [83]. Due to special immune modifications that reduce harmful inflammation, bats can withstand infections. They prevent excessive tissue damage during infection by exhibiting reduced function of important components like caspase-1 and IL-1β, as well as inhibited activation of the NLR family pyrin domain-containing 3 (NLRP3) inflammasome [83]. Certain species, including *Pteropus alecto*, appear to be able to control viruses without causing damaging inflammation because they maintain controlled expression of type I interferons [83]. Bats also have abnormalities in the Stimulator of Interferon Genes (STING) system and lack several DNA sensors, such as the pyrin and hematopoietic, interferon-inducible nuclear (HIN) domain-containing (PYHIN) genes, which lessens the overreaction to flight-induced DNA damage that causes inflammation [83]. Their improved antioxidant and DNA repair systems also reduce oxidative stress-induced inflammation [83]. These characteristics work together to allow bats to carry large virus loads without becoming ill [83].

Through redundancy and diversification of additional factors, the Zf immune system remains robust despite the apparent low sensitivity or changed function of TLR4. It has been suggested that these receptors might not detect traditional PAMPs, but rather endogenous damage-associated molecular patterns (DAMPs), danger signals (alarmins) like High Mobility Group Box 1 (HMGB1). This theory is supported by recent neurotoxic stress studies involving Zf, showing increased expression of *HMGB1* and the *HMGB1/TRL4*/*NF-κB* pathway in response to the neurotoxin 1-methyl-4-phenyl-1,2,3,6-tetrahydropyridine (MPTP) [84]. This implies that rather than microbial products like LPS, Zf TLR4 may mainly be involved in the detection of DAMPs, which trigger inflammatory pathways in response to cellular injury [84]. Reduced TLR4-mediated LPS detection can be compensated for by the broader repertoires of Zf TLR isoforms, as well as other PRRs such as NLRs and PGRPs. Without depending exclusively on TLR4, this diversity of receptors offers an adaptable and robust immune recognition system that can identify a wide range of pathogens and microbial compounds. By avoiding the dangers of overactivating a single receptor pathway, this redundancy guarantees that Zf can develop efficient immunological responses. The benefit of preserving a strong and flexible immune system that can successfully regulate inflammatory reactions is likely reflected in the evolutionary conservation of these many LPS-sensing mechanisms in aquatic vertebrates.

## 6. Conclusions

This review emphasizes that, in stark contrast to mammals, where the TLR4/MD-2 complex is essential for both LPS detection and inflammatory signaling, Zf largely mount an immune response to LPS through TLR4-independent pathways. Rather than serving as conventional LPS receptors, Zf TLR4 paralogs appear to have a context-dependent or a limited role in LPS sensing, potentially acting as negative regulators of the MyD88-dependent NF-κB signaling pathway. Although the exact molecular mechanisms underlying Zf LPS responses remain unclear, various key immunological mediators, including NOD1 and complement C3, as well as other as-yet-unidentified PRRs, are likely to contribute to these responses.

An ancestral, low-affinity LPS recognition capability may be evolutionarily conserved, as evidenced by the recent discovery of *ly96* and the demonstration that TLR4ba can form a functional complex with ly96 to activate NF-κB, albeit only under artificial conditions involving mammalian CD14 and elevated LPS concentrations. The absence of a clearly defined Zf CD14 homolog, along with the complete loss of TLR4 in the majority of fish species, raises significant questions about the biological significance of this pathway in vivo.

These results highlight important information gaps and the pressing need for more functional and genetic research, including thorough transcriptome and proteome profiling of Zf after LPS exposure and clustered regularly interspaced short palindromic repeats (CRISPR)-mediated KOs of potential receptors. Deciphering the evolutionary diversification of LPS-sensing mechanisms will also require comparative immunological investigations across teleost species. In the end, this kind of study will help us better understand the evolutionary history of innate immunity as well as the special methods used by aquatic vertebrates to recognize and react to bacterial endotoxins.

The Zf provides a versatile and efficient platform for studying immune systems and assessing new treatment modalities, especially through live imaging, genetic alteration, and high-throughput drug screening. NLRs, complement factors, inflammasome components, and PGRPs are among the intriguing targets for selective immunomodulation that have been identified through the study of different LPS-sensing systems in Zf. By concentrating on these pathways, pathogen removal or pathological inflammation can potentially be enhanced without resulting in the widespread immunosuppression that TLR4 antagonists in mammals frequently do [85,86]. These targets can be functionally validated using transgenic and CRISPR/Cas9-modified Zf, and preclinical testing of immunotherapies is further supported by Zf patient-derived xenograft models, known as “zAvatars” [87]. These zAvatars have shown prognostic usefulness for patient treatment outcomes and progression by transferring patient tumor cells into larvae, enabling quick evaluation of medication responses, including checkpoint progression-free survival [87]. Additionally, real-time imaging of immune cell dynamics and quick assessment of potential chemicals influencing cytokine production, oxidative stress, and cell migration are made possible by Zf larval inflammation models, especially transgenic lines containing fluorescently labeled neutrophils and macrophages [74,88,89]. All things considered, employing Zf for therapeutic testing and mechanistic research has the potential to accelerate the identification of specific immunomodulatory targets, facilitate the logical development of combination treatments, and open the door to personalized medicine techniques. Thus, the Zf is an affordable, high-throughput platform for translational research in immuno-oncology, inflammatory diseases, infections, and immune-mediated illnesses.

## Figures and Tables

**Table 1:** Zebrafish (Zf, *D. rerio*) as a model organism: important research areas and contributions.

Research Area	Findings and Contributions	Key Reference(s)
Immunology	Nanoparticles were tested in Zf embryos, which demonstrated increased innate immune activation.	[6]
Cellular Metabolism and Redox Biology	An Apollo-NADP^+^ biosensor was developed to track real-time NADPH/NADP^+^ dynamics in Zf embryos.	[7]
Cancer Research	Adult transparent Zf were used to study RAS melanoma cell xenotransplantation, tumor engraftment, proliferation, and metastases.	[8]
Toxicology	Genetically engineered Zf were used for detecting toxicant exposure. Their rapid life cycle allows for quick assessment of exposure effects.	[9,10,11]
Neuroscience and Neurodegeneration	Zf were used as an Alzheimer’s disease model due to genetic similarity, simple nervous system, and transparent embryos.	[12,13,14]

## Data Availability

The datasets used and analyzed during the current study are available from the corresponding author upon request.
